# A-Site Cation Size Effect on Structure and Magnetic Properties of Sm(Eu,Gd)Cr_0.2_Mn_0.2_Fe_0.2_Co_0.2_Ni_0.2_O_3_ High-Entropy Solid Solutions

**DOI:** 10.3390/nano12010036

**Published:** 2021-12-23

**Authors:** Denis A. Vinnik, Vladimir E. Zhivulin, Evgeny A. Trofimov, Svetlana A. Gudkova, Alexander Yu. Punda, Azalia N. Valiulina, Maksim Gavrilyak, Olga V. Zaitseva, Sergey V. Taskaev, Mayeen Uddin Khandaker, Amal Alqahtani, David A. Bradley, M. I. Sayyed, Vitaliy A. Turchenko, Alex V. Trukhanov, Sergei V. Trukhanov

**Affiliations:** 1 Laboratory of Single Crystal Growth, South Ural State University, 76, Lenin Av., 454080 Chelyabinsk, Russia; denisvinnik@gmail.com (D.A.V.); zhivulinve@susu.ru (V.E.Z.); tea7510@gmail.com (E.A.T.); svetlanagudkova@yandex.ru (S.A.G.); pundaai@susu.ru (A.Y.P.); an_valiulina@mai.ru (A.N.V.); gavrilyak.maksim@yandex.ru (M.G.); nikonovaolga90@gmail.com (O.V.Z.); s.v.taskaev@gmail.com (S.V.T.); va_turchenko@mail.ru (V.A.T.); truhanov86@mail.ru (A.V.T.); 2 Centre for Applied Physics and Radiation Technologies, School of Engineering and Technology, Sunway University, Bandar Sunway 47500, Selangor, Malaysia; mayeenk@sunway.edu.my (M.U.K.); d.a.bradley@surrey.ac.uk (D.A.B.); 3 Department of Basic Sciences, Deanship of Preparatory Year and Supporting Studies, Imam Abdulrahman Bin Faisal University, P.O. Box 1982, Dammam 34212, Saudi Arabia; amalqahtani@iau.edu.sa; 4 Centre for Nuclear and Radiation Physics, Department of Physics, University of Surrey, Guildford GU2 7XH, Surrey, UK; 5 Department of Physics, Faculty of Science, Isra University, Amman 11622, Jordan; dr.mabualssayed@gmail.com; 6 Department of Nuclear Medicine Research, Institute for Research and Medical Consultations (IRMC), Imam Abdulrahman Bin Faisal University, Dammam 31441, Saudi Arabia; 7 Laboratory of Neutron Physics, Joint Institute for Nuclear Research, 6, Joliot-Curie Str., 141980 Dubna, Russia; 8 Donetsk Institute of Physics and Technology Named after O.O. Galkin of the NASU, 46, Nauki Av., 03680 Kiev, Ukraine; 9 Laboratory of Magnetic Films Physics, SSPA “Scientific and Practical Materials Research Centre of NAS of Belarus”, 19, P. Brovki str., 220072 Minsk, Belarus; 10 L.N. Gumilyov Eurasian National University, 2, Satpayev str., Nur-Sultan 010000, Kazakhstan

**Keywords:** transition metals, crystal structure, high entropy perovskites, magnetic properties, indirect superexchange interactions

## Abstract

Three high-entropy Sm(Eu,Gd)Cr_0.2_Mn_0.2_Fe_0.2_Co_0.2_Ni_0.2_O_3_ perovskite solid solutions were synthesized using the usual ceramic technology. The XRD investigation at room temperature established a single-phase perovskite product. The Rietveld refinement with the FullProf computer program in the frame of the orthorhombic Pnma (No 62) space group was realized. Along with a decrease in the V unit cell volume from ~224.33 Å^3^ for the Sm-based sample down to ~221.52 Å^3^ for the Gd-based sample, an opposite tendency was observed for the unit cell parameters as the ordinal number of the rare-earth cation increased. The average grain size was in the range of 5–8 μm. Field magnetization was measured up to 30 kOe at 50 K and 300 K. The law of approach to saturation was used to determine the M_s_ spontaneous magnetization that nonlinearly increased from ~1.89 emu/g (Sm) up to ~17.49 emu/g (Gd) and from ~0.59 emu/g (Sm) up to ~3.16 emu/g (Gd) at 50 K and 300 K, respectively. The M_r_ residual magnetization and H_c_ coercive force were also determined, while the SQR loop squareness, k magnetic crystallographic anisotropy coefficient, and H_a_ anisotropy field were calculated. Temperature magnetization was measured in a field of 30 kOe. ZFC and FC magnetization curves were fixed in a field of 100 Oe. It was discovered that the T_mo_ magnetic ordering temperature downward-curve decreased from ~137.98 K (Sm) down to ~133.99 K (Gd). The spin glass state with ferromagnetic nanoinclusions for all the samples was observed. The <D> average and D_max_ maximum diameter of ferromagnetic nanoinclusions were calculated and they were in the range of 40–50 nm and 160–180 nm, respectively. The mechanism of magnetic state formation is discussed in terms of the effects of the A-site cation size and B-site poly-substitution on the indirect superexchange interactions.

## 1. Introduction

Complex oxides based on transition metal cations have been the object of close research because of their fundamental importance and applied value for more than 70 years [1]. Among the specified class of compounds, the most promising are oxides with a perovskite structure [2]. This is due to their rich properties that allow them to be used as multifunctional materials [3]. The so-called multiferroics [4] are of great practical importance; multifunctional compounds simultaneously possessing two or more ferroic orderings are amenable to controlled change by coupled fields [5]. This behavior is especially important when solving problems of the induction, conversion, accumulation, and storage of electromagnetic energy [6].

The brightest representatives of perovskites are the BaTiO_3_ barium titanate with well-known ferroelectric properties [7], the doped Re_1-x_A_x_Mn_1-y_B_y_O_3_ rare-earth orthomanganites with colossal magnetoresistance, and a diverse magnetic phase state [8]. The electrical and magnetic properties of the manganites are determined by the Mn^3+^-O^2−^-Mn^3+^ indirect superexchange interactions [9,10] associated with virtual transitions of the localized e_g_ electrons between Mn^3+^ cations [11] and with the special role of Jahn–Teller distortions of oxygen octahedra around the Mn^3+^ cation [12]. The intensity of the Mn^3+^-O^2−^-Mn^3+^ indirect superexchange interactions constantly increases with the 〈Mn^3+^-O^2−^-Mn^3+^〉 average bond angle to 180 degrees and with the decrease in the 〈Mn^3+^-O^2−^〉 average bond length [13].

The degree of structural disorder in condensed matter determines such a thermodynamic parameter as entropy [14]. The most effective change in entropy depends on the cationic composition and location in the unit cell. Until recently, entropy was not considered as effective as enthalpy in materials development [15].

As is well known, the phase stability of a solid solution is determined by the change in the Gibbs free energy [16]. This change consists of a positive change in the mixing enthalpy and a negative change in the mixing entropy multiplied by the absolute temperature. With an increase in the mixing entropy, the change in the Gibbs free energy decreases, which leads to the stabilization of the phase state of the solid solution. The mixing entropy of a solid solution is determined by the number of mixing elements, the concentration of mixing elements, and the absolute temperature [17]. For each specific temperature, the value of entropy is maximum for a solid solution with an equal concentration of mixing elements, when the number of such elements rises upward [18]. It is generally accepted from practice that solid solutions containing more than five elements of the same type with a concentration of 20% should be classified as high-entropy ones [19]. It follows from this that the condition for the existence of a high-entropy solid solution is that the entropy exceeds the value of 1.61 × R, where R is the universal gas constant [20]. Such compounds demonstrate a unique combination of composition, structure, and physicochemical properties [21]. The most popular among high-entropy compounds are high-entropy oxides [22].

For the oxide perovskites, the production of high-entropy solid solutions is reduced to the substitution of at least five components in the A or B sublattice [23]. Such substitutions have two consequences: first, a structural consequence, where there is a change in the average radius of the corresponding sublattice and the associated possible removal of the cooperative static Jahn–Teller effect in the case of substitution in the B sublattice for the orthomanganites [24]; and secondly, an exchange consequence, where there is the weakening of exchange interactions associated with magnetic dilution [25]. Therefore, in the case of obtaining a high-entropy solid solution of perovskite with multicomponent substitution in the B sublattice, one should expect, along with an increase in phase stability, a weakening of the magnetic properties.

In the present paper, the Sm(Eu,Gd)Cr_0.2_Mn_0.2_Fe_0.2_Co_0.2_Ni_0.2_O_3_ high-entropy solid solutions were prepared by the usual ceramic technology and were investigated by XRD, SEM, and SQUID magnetometry. Effects of the A-site cation size and B-site poly-substitution on the Mn^3+^(Cr^3+^, Fe^3+^, Co^3+^, Ni^2+^)-O^2−^-Mn^3+^(Cr^3+^, Fe^3+^, Co^3+^, Ni^2+^) indirect superexchange interactions and magnetic properties were also studied.

## 2. Materials and Methods

### Synthesis and Characterization

The samples of the three compositions of Sm(Eu,Gd)Cr_0.2_Mn_0.2_Fe_0.2_Co_0.2_Ni_0.2_O_3_ with the perovskite structure were synthesized using the usual ceramic technology. The high-purity (99.999%) rare-earth oxides of Sm_2_O_3_, Eu_2_O_3_, and Gd_2_O_3_, as well as the high-purity (99.999%) 3d metal oxides of Cr_2_O_3_, Mn_2_O_3_, Fe_2_O_3_, CoO, and NiO (CAS 1313-99-1, Luoyang Tongrun Nano Technology Co. Ltd., Luoyang, China), were used as the starting reagents. The chemical reaction of synthesis may be written as:

(1) $$0.1{\mathrm{Cr}}_{2}O_{3}+0.1{\mathrm{Mn}}_{2}O_{3}+0.1{\mathrm{Fe}}_{2}O_{3}+0.2\mathrm{CoO}+0.2\mathrm{NiO}+0.5\mathrm{Sm}{\left(\mathrm{Eu},\mathrm{Gd}\right)}_{2}O_{3}+0.2O_{2}\to \mathrm{Sm}\left(\mathrm{Eu},\mathrm{Gd}\right){\mathrm{Cr}}_{0.2}{\;\mathrm{Mn}}_{0.2}{\mathrm{Fe}}_{0.2}{\mathrm{Co}}_{0.2}{\mathrm{Ni}}_{0.2}O_{3}$$

The mixed powders of the required composition were grounded during 3 h in a ball mill with added alcohol. After this, the mixes were pressed in the form of 0.5 cm × 1 cm cylindrical pellets under a pressure of 3 tons/cm^2^. Further, the samples were calcined at 1400 °C in air during 5 h followed by slow cooling down to room temperature [26].

XRD (Rigaku Ultima IV) (Rigaku Europe SE, Neu-Isenburg, Germany) with Cu-K_α_ radiation was used to check the phase content and calculate the unit cell parameters at room temperature. The angular scanning range was 20–90° with a rate of 1°/min. The Rietveld refinement [27] of XRD data in the frame of the Fullprof computer program (PROGRAM FullProf.2k (Version 7.40-March2021-ILL JRC), https://www.ill.eu/sites/fullprof/php/programs8809.html?pagina=FullProf (accessed on 18 December 2021)) [28] was used. The chemical composition was checked by EDX (Oxford INCA X-max 80) (Oxford Instruments, Moscow, Russian Federation). SEM was applied to obtain the microstructure images. SmartSEM (SmartSEM V05.06) software (Carl Zeiss SMT GmbH, Oberkochen, Germany) was used to treat three SEM images and to determine the average particle size for each sample [29].

The magnetization measurements were realized using a SQUID magnetometer [30]. The M(H) field magnetization loop was obtained in the range of 30–−30 kOe at 50 K and 300 K. The law of approach to saturation [31] was used to determine the M_s_ spontaneous magnetization. The M_r_ residual magnetization, SQR = M_r_/M_s_ squareness of the loop, and H_c_ coercive force were determined. The k magnetic crystalline anisotropy coefficient and H_a_ anisotropy field were calculated. The temperature magnetization was measured in the range of 50–300 K in a field of 30 kOe. The point of minimum of the dM/dT(T) magnetization derivative or zero of the d^2^M/dT^2^(T) second magnetization derivative was taken as the T_mo_ magnetic ordering or T_c_ Curie temperature [32].

## 3. Results and Discussion

### 3.1. Crystal Structure

**Figure 1:**
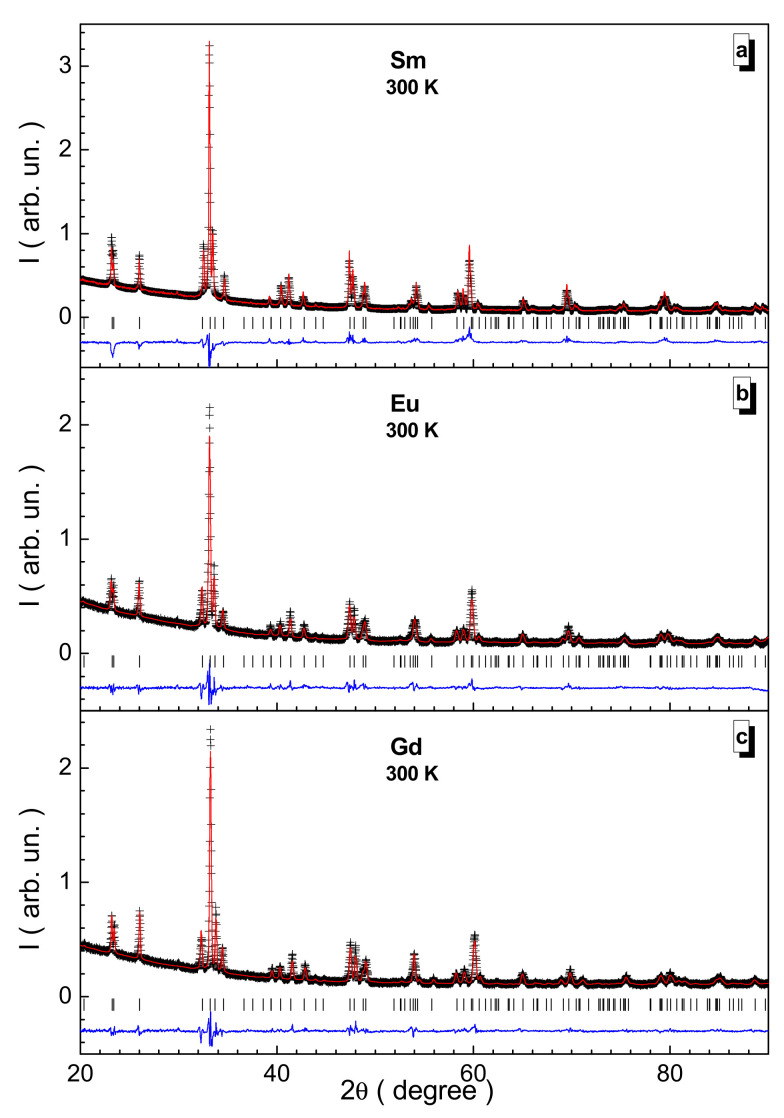
Rietveld-refined XRD pattern for the Sm- (**a**), Eu- (**b**), and Gd-based (**c**) samples. The experimental data (cross), fitting curve (red line), theoretical Bragg positions (vertical bar), and difference curve (blue line) are presented.

The phase purity and refinement of the unit cell parameters of the three obtained solid solutions were realized using X-ray powder diffraction at room temperature. The initial experimental data, as well as the results of the Rietveld fitting using the FullProf program, are shown in Figure 1. It is clearly seen that the results of matching the structural parameters within the Pnma (No. 62) space group give satisfactory results, as evidenced by the shape of the difference curve. When refining the unit cell parameters and ion coordinates, the χ^2^ fitting goodness parameter does not exceed 2.5.

**Figure 2:**
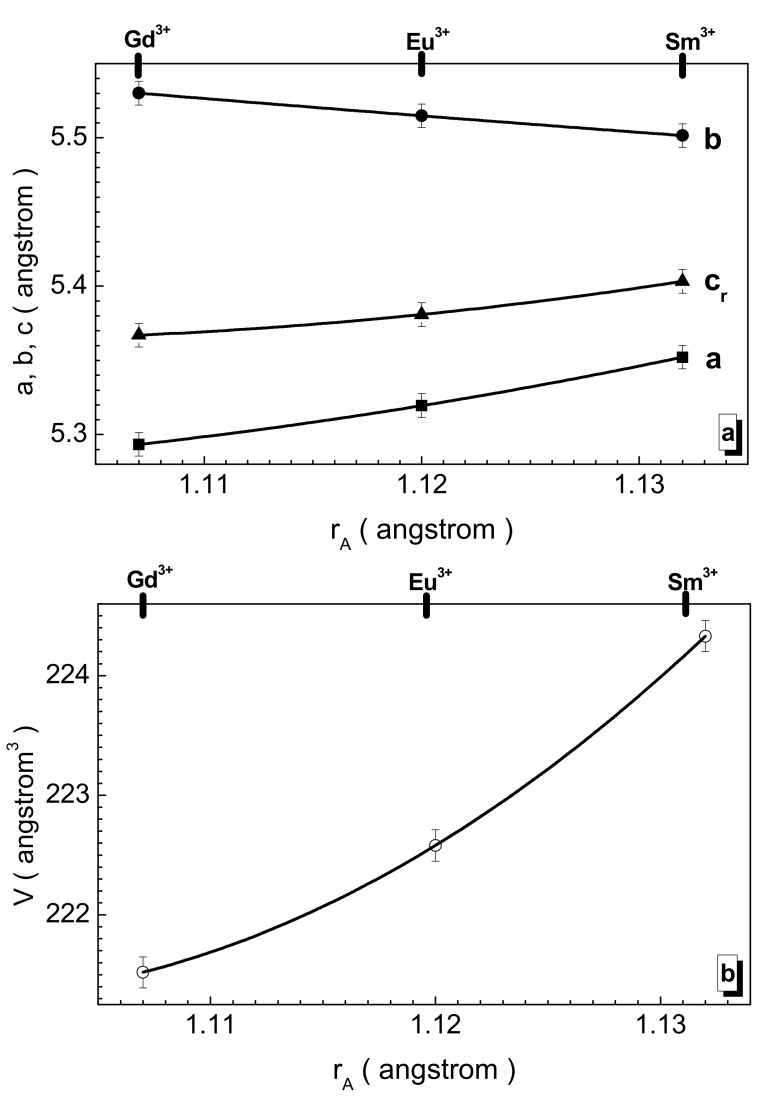
Dependence of unit cell parameters (**a**) a (full rectangle), b (full circle), and c/√2 = c_r_ (full up-triangle), as well as unit cell volume (**b**) V (open circle) vs. A-site cation radius for the obtained samples. Solid line is the second-order polynomial interpolation and it is an eye guide.

The unit cell parameters change monotonically and almost linearly. However, they have a multidirectional character of change with an increase in the serial number of the rare-earth cation. Thus, the *a* and *c* parameters decrease, and the *b* parameter increases (see Figure 2a). The maximum value of the *a* parameter is fixed for the samarium sample and is ~5.352 Å. The maximum value of the *b* parameter is fixed for the gadolinium sample and it is ~5.530 Å. The relative decreases in the *a* and *c* parameters are ~1.1 and ~0.7%, respectively, when going from the Sm^3+^ cation to the Gd^3+^ cation, while the relative increase in the *b* parameter is ~0.5%. The maximum V unit cell volume is observed for the samarium sample and is ~224.33 Å^3+^. It decreases by ~1.3% when going to the Gd^3+^ cation.

It should be noted here that the Mn^3+^ cations in the octahedral environment of the O^2−^ oxygen anions are the Jahn–Teller ones, as their doubly degenerated e_g_ electron orbital is half-filled. All the cations participating in the formation of the studied solid solutions have an oxidation state of 3+. This follows from the laws of conservation of electric charge and electroneutrality of macroscopic bodies when the solid solution is stoichiometric. Of all the cations present with the oxidation state 3+ in the octahedral anionic coordination, only the Mn^3+^ manganese cation is the Jahn–Teller one.

Among the considered cations, the Jahn–Teller cations in an octahedral environment can also be cations of a triply charged Ni^3+^ nickel cation, which has 7 d electrons, in a high-spin state, and a triply charged Co^3+^ cobalt cation, which has 6 d electrons, in an intermediate spin state. Strictly speaking, it is theoretically possible to admit the observation of various combinations of oxidized states for the considered 3d transition metal cations at anionic stoichiometry, such as 3+-3+-3+-2+-4+ and 3+-2+-4+-2+-4+. However, the nickel cation, when synthesized in an air atmosphere, has a stable tendency to form the 2+ oxidized state. Then, for charge compensation, the transition of other used cations into the 4+ oxidized state is required. In this case, this can most likely be observed for the manganese cation. However, as the value of the electron affinity for the considered chemical elements is close to each other, it is possible to admit the development of such a situation only in a certain approximation. Moreover, each type of cation can have different oxidation states, i.e., the so-called charge disproportionation state can be observed. This further complicates the problem and it is clear that its solution requires additional theoretical and experimental studies.

Therefore, complex manganese oxides with a perovskite-like structure are often characterized by the Jahn–Teller effect. The latter manifests itself in the polar distortion of the oxygen octahedron. Two bond lengths become longer or shorter than the remaining four. The specific behavior and distortion of the unit cell observed in the samples under study is quite well explained by the size factor of the rare-earth cations filling the A positions in the perovskite structure and the absence of the cooperative static Jahn–Teller distortions. The absence of the cooperative static Jahn–Teller distortions is indicated by the observed O-orthorhombic symmetry of the unit cell. This type of distortion is characterized by a certain sequence of the unit cell parameters, which is as follows (a < c/√2 < b) [33]. In the presence of the cooperative static Jahn–Teller distortions, the sequence of the unit cell parameters changes (c/√2 < a ≤ b), which indicates the formation of the so-called O^/^-orthorhombic symmetry [34].

### 3.2. Microstructure

**Figure 3:**
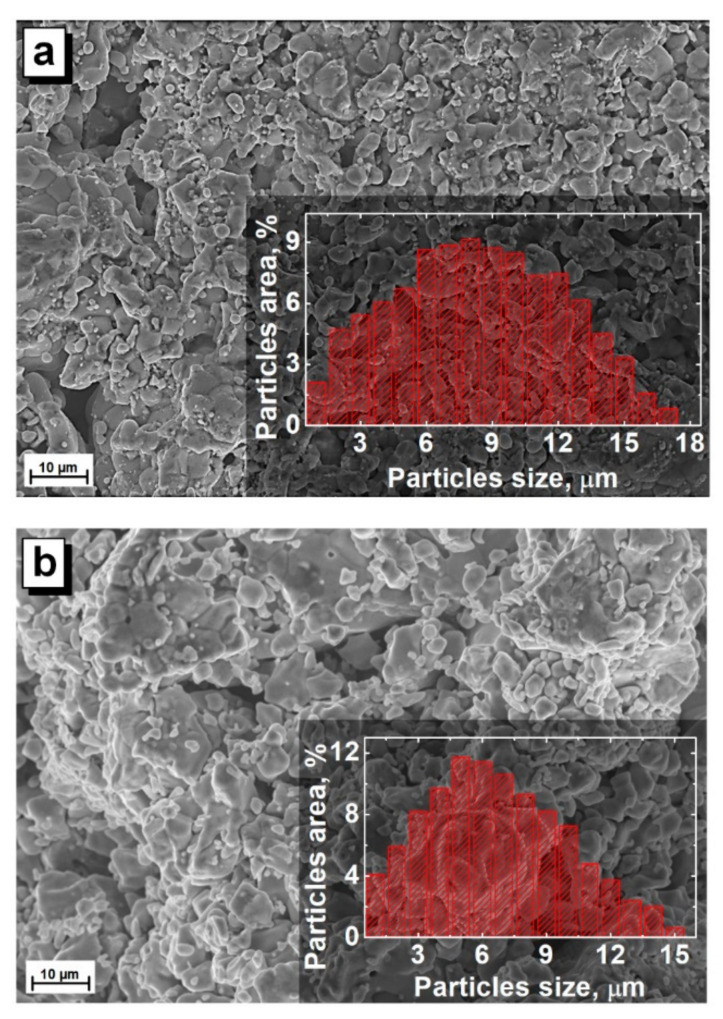

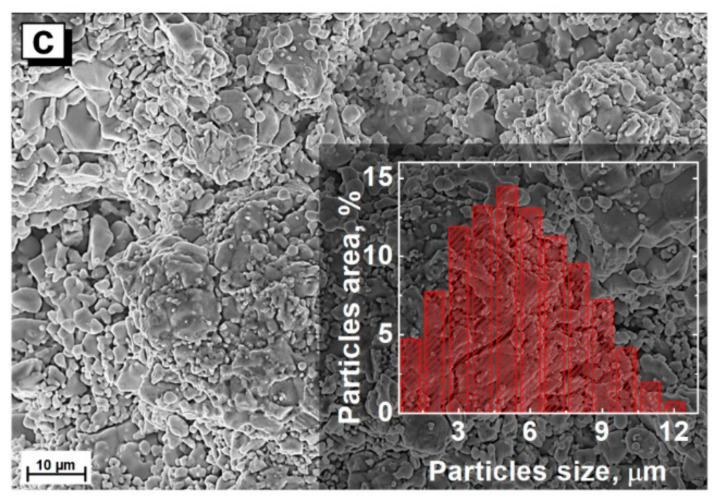
SEM image for the Sm- (**a**), Eu- (**b**), and Gd-based (**c**) samples. Inset demonstrates the particle size distribution.

The surface images of the obtained ceramic samples presented in Figure 3 by the SEM method demonstrate a dense microstructure of granules of irregular shape and different sizes. As the serial number of the rare-earth cation increases, the maximum grain size decreases. Thus, for a samarium sample, the maximum grain size reaches ~17 µm; for a europium sample, it reaches ~16 µm, while, for a gadolinium sample, it reaches only ~13 µm.

Grains with sizes above these values were not observed. The relative fraction of grains from their total number with minimum sizes up to 1 μm was ~2, ~4, and ~5% for samarium, europium, and gadolinium samples, respectively, while the relative fraction of grains with maximum sizes reached only 1%. The maximum relative fraction of grains, which was ~9, ~12, and ~15% for samarium, europium, and gadolinium samples, reached sizes of ~8, ~5, and ~5 µm, respectively. The average grain size was in the range of 5–8 μm.

### 3.3. Magnetic Properties

**Figure 4:**
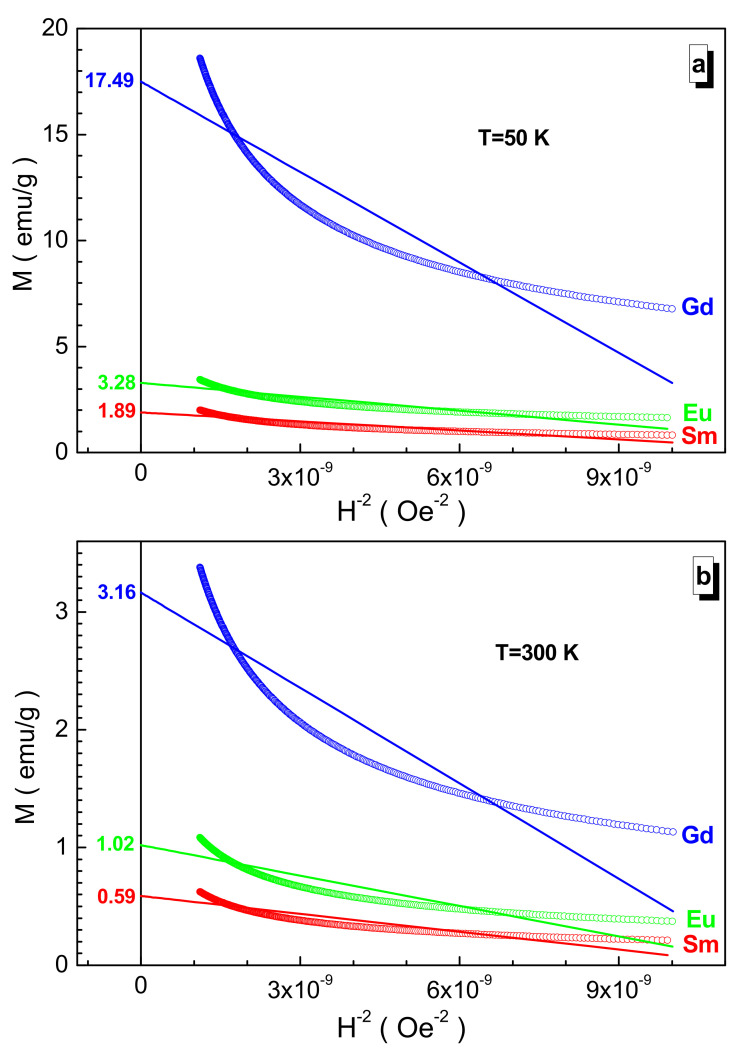
Dependence of magnetization vs. inverse square of the field for the Sm- (red circle), Eu- (green circle), and Gd-based (blue circle) samples at 50 K (**a**) and 300 K (**b**). Straight line is the linear extrapolation.

From Figure 4, two main features are clearly visible. The first is that with an increase in the serial number of the rare-earth cation, the spontaneous magnetization increases; moreover, for the gadolinium sample, it increases significantly. The second is that with increasing temperature, the spontaneous magnetization decreases accordingly.

These two features are explained by the enhancement of the Mn^3+^(Cr^3+^, Fe^3+^, Co^3+^, Ni^2+^)-O^2—^Mn^3+^(Cr^3+^, Fe^3+^, Co^3+^, Ni^2+^) indirect superexchange interactions as a result of a decrease in the 〈Mn^3+^(Cr^3+^, Fe^3+^, Co^3+^, Ni^2+^)-O^2−^〉 average bond length of B-cations and an increase in the disordering action of thermal energy. The minimum value of the spontaneous magnetization is observed for the samarium sample, which is ~1.89 emu/g at 50 K and ~0.59 emu/g at 300 K. This value increases when going to the Gd^3+^ cation by ~825% at 50 K and by ~436% at 300 K.

**Figure 5:**
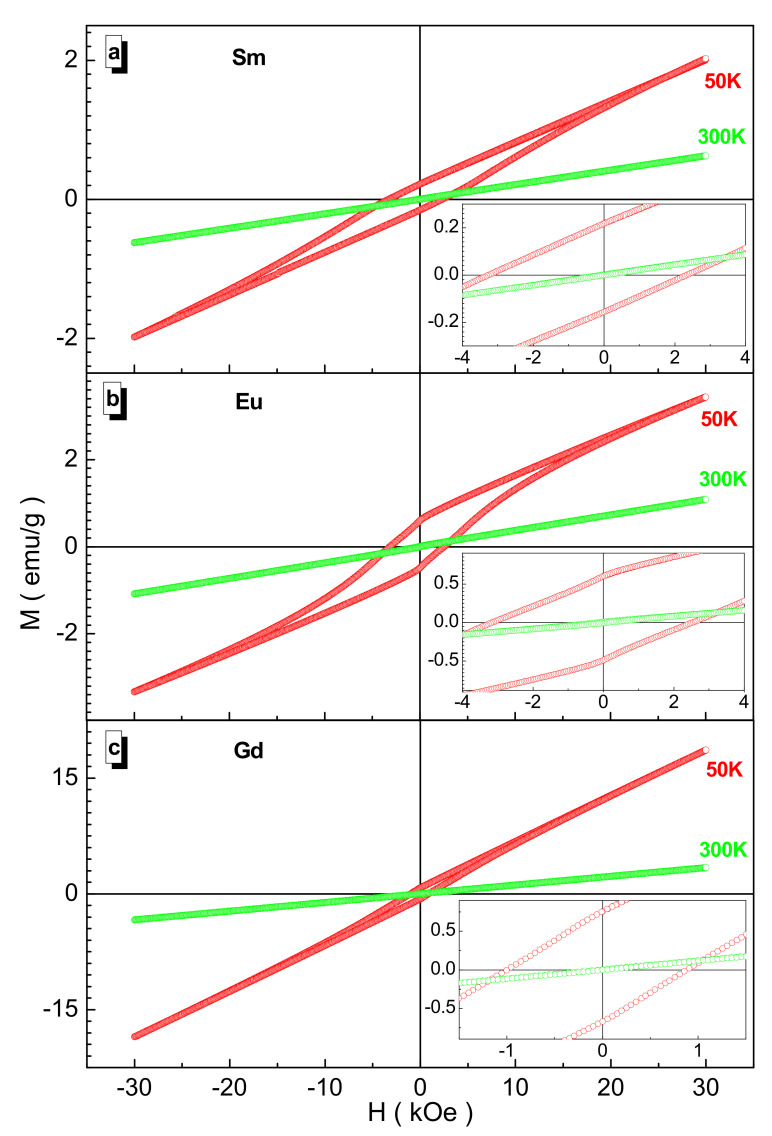
Field magnetization for the Sm- (**a**), Eu- (**b**), and Gd-based (**c**) samples at 50 K (red circle) and 300 K (green circle). Inset demonstrates the field magnetization on a larger scale.

From the shape of the hysteresis loop in Figure 5, it can be concluded that for all the samples, there is a noticeable residual magnetization and coercive force, and with increasing temperature, they noticeably decrease. The maximum value of the residual magnetization of ~0.72 emu/g is observed for the gadolinium sample at 50 K, which decreases by ~73% when going to the samarium sample. The maximum value of the coercive force of ~2.81 Oe is observed for the samarium sample at 50 K, which decreases by ~67% upon passing to the gadolinium sample. This is due to the disordering of local spins by thermal energy and the destruction of domain walls.

**Figure 6:**
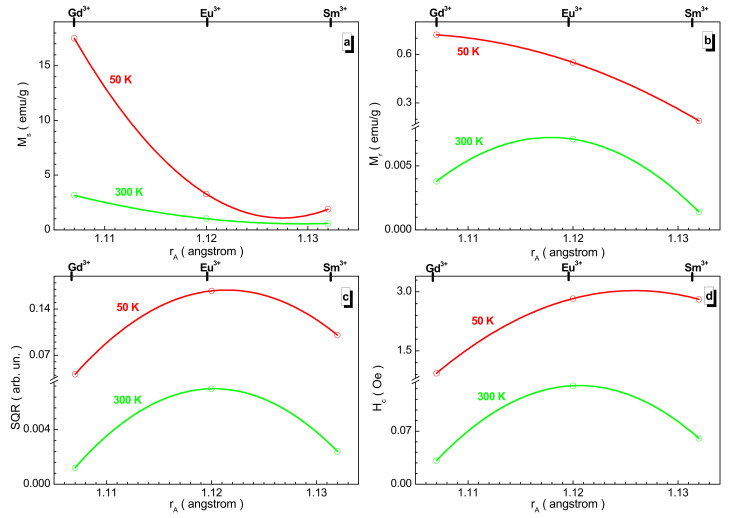
Dependence of the M_s_ spontaneous magnetization (**a**), M_r_ residual magnetization (**b**), SQR = M_r_/M_s_ squareness of the loop (**c**), and H_c_ coercive force (**d**) vs. A-site cation radius size at 50 K (red circle and line) and 300 K (green circle and line) for the obtained samples. The solid line is the second-order polynomial interpolation and it is an eye guide.

The generalized dependences of the magnetic parameters taken from the field magnetization depending on the rare-earth cation size are shown in Figure 6. Almost all of these parameters do not change monotonically, that is, they have an inflection point with the exception of the spontaneous magnetization at both temperatures and residual magnetization at 50 K. All the low-temperature parameters are dominant. The maximum loop squareness observed for the europium sample is small and tends to ~0.17 at 50 K.

**Figure 7:**
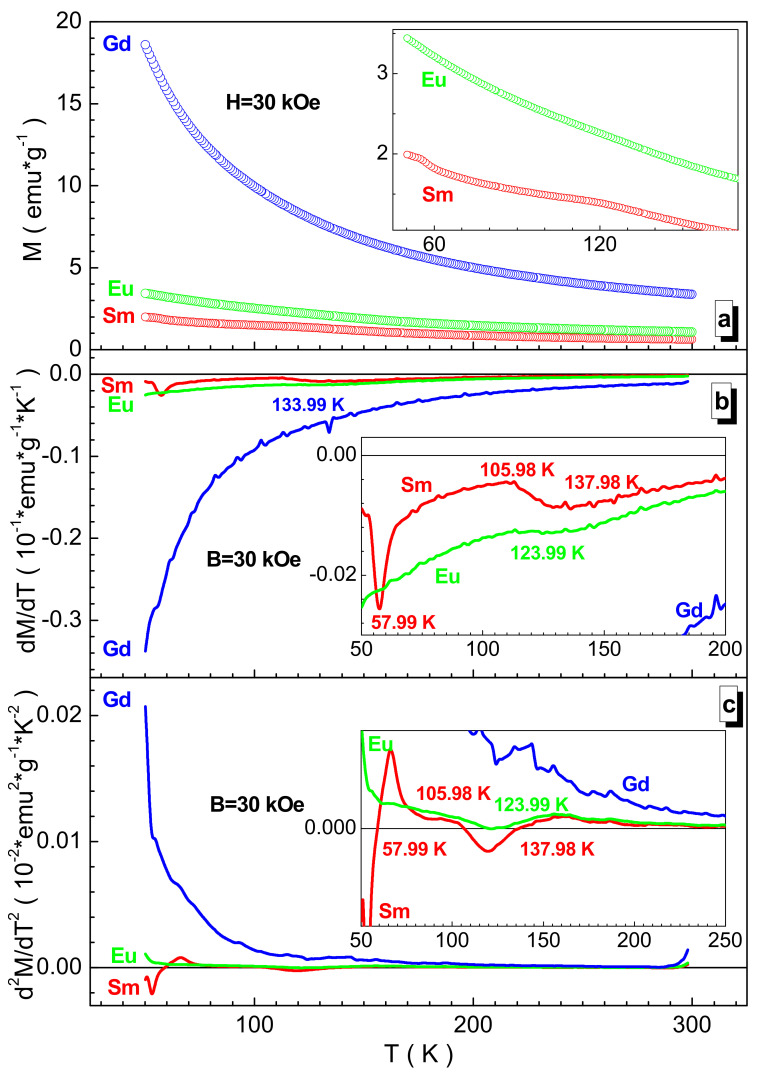
M(T) temperature magnetization (**a**), dM/dT(T) first magnetization derivative (**b**), and d^2^M/dT^2^(T) second magnetization derivative for the Sm- (red circle), Eu- (green circle) (**c**), and Gd-based (blue circle) samples in 3 T. Inset demonstrates the temperature magnetization on a larger scale.

Figure 7 shows the temperature dependences of magnetization in a strong field for the studied samples. They decrease almost monotonously with the field. Only for samarium and europium samples is an anomaly observed in the region of ~100–140 K. Such a constant decrease in magnetization means a diffuse phase transition to the paramagnetic state, which is characteristic of partially frustrated spin systems when clusters with different degrees of local spin ordering are present. It is the gradual disordering of these clusters that causes the smearing of the magnetic phase transition. To characterize this state, the magnetic ordering temperature was used, which is the point of maximum decrease in the corresponding function graph [35]. This magnetic ordering temperature was determined by the differential method from the extrema of the dM/dT(T) first derivative or from the zeros of the d^2^M/dT^2^(T) second derivative of the M(T) magnetization. The maximum T_mo_ magnetic ordering temperature of ~134 K was recorded for the gadolinium sample, and the minimum T_mo_ of ~124 K was found for the europium sample. The anomalies for the samarium sample at ~58 K and ~106 K are most likely associated with the disordering of antiferromagnetic clusters formed by indirect superexchange interactions from the Mn^3+^(Cr^3+^, Fe^3+^, Co^3+^, Ni^2+^)-O^2^-Ni^2+^ (Mn^3+^, Cr^3+^, Fe^3+^, Co^3+^) set [36].

The orthoferrites have the highest magnetic ordering temperature among all the complex 3d transition metal oxides used in this work. The SmFeO_3_ samarium orthoferrite is a canted G-type antiferromagnet and their T_N_ Néel temperature reaches ~680 K [37]. It is interesting that the disordering temperature of the competing exchange between the f- and d-electrons of the Sm^3+^ samarium and Fe^3+^ iron cations reaches ~140 K [38]. With an increase in the serial number of the rare-earth cation, the samples are characterized by a decrease in the magnetic ordering temperature [39] with the dominant Fe^3+^-O^2^-Fe^3+^ antiferromagnetic interactions and weak ferromagnetism due to the low-temperature ordering of the rare-earth magnetic sublattice [40].

The SmMnO_3_ samarium manganite is an inhomogeneous A-type antiferromagnet [41] with the magnetic ordering temperature just below 90 K [42]. As the serial number of the rare-earth cation increases, the magnetic ordering temperature gradually decreases [43]. For the EuMnO_3_ europium manganite, it is only T_mo_~57 K [44]. For the GdMnO_3_ gadolinium manganite, it is T_mo_~51 K [45].

The perovskites based on the remaining rare-earth cations have even weaker magnetic properties. Thus, the magnetic state of the SmCoO_3_ samarium orthocobaltite is complicated by the presence of spin phase separation under various external conditions. Near the ~500 K metal–dielectric transition, it is known [46]. This is explained by a change in the spin state of the Co^3+^ cobalt cation [47], which is determined by such parameters as the local crystal structure, the composition of substituents, and applied external fields such as thermal, baric, magnetic, and electric. Regardless of the crystal structure phase, the SmCoO_3_ samarium orthocobaltite becomes metallic, as there is no forbidden band at the Fermi level. Thus, the orthorhombic phase becomes metallic when it has an A-type or C-type antiferromagnet structure, but remains semiconducting when the compound has a G-type antiferromagnet structure. Therefore, the metal–dielectric transition in the SmCoO_3_ samarium orthocobaltite is not associated with the magnetic structure [48]. As the serial number of the rare-earth cation increases, the magnetic ordering temperature for the orthorhombic cobaltites decreases. Below room temperature, the main contribution to the magnetization is made by the paramagnetism of the rare-earth cations. The SmCoO_3_ samarium orthocobaltite and GdCoO_3_ gadolinium orthocobaltite are significantly different in magnetic terms. Such behavior is associated with differences in the structure of the electron shells of the rare-earth cations. For the GdCoO_3_ gadolinium orthocobaltite, the magnetic behavior is close to the behavior of a set of free Gd^3+^ cations, while for the SmCoO_3_ samarium orthocobaltite, a behavior more characteristic for bound cations is observed due to the fact that the ground state of Sm^3+^ is split by the crystal field of anions [49].

For the SmCrO_3_ samarium orthochromite, three different magnetic phases are observed with decreasing temperature: first, there is an uncompensated canted antiferromagnetic structure that appears below the T_N_ ≈ 191 K Néel temperature; second, a collinear antiferromagnetic structure that appears below ~10 K; and third, a nonequilibrium configuration with interacting phases in the vicinity of a spin-reorientation phase transition in the temperature range of 10 K ≤ T ≤ 40 K [50]. With an increase in the serial number of the rare-earth cation in such canted antiferromagnetic structures as the orthocromites with a weak ferromagnetic component, the Néel temperature gradually decreases in the range from T_N_ ≈ 288 K for the LaCrO_3_ to T_N_ ≈ 112 K for the LuCrO_3_ [51].

It is difficult to characterize the magnetic structure and T_mo_ magnetic ordering temperature of the rare-earth orthonickelates, as these compounds are unstable in bulk due to the high oxygen pressure required to stabilize the nickel cation in the 3+ oxidation state [52]. Such stabilization can be achieved and maintained in multilayer thin-film structures, where each of the individual layers in their bulk form is unstable [53]. Such multilayer structures of the orthonickelates are characterized by complex antiferromagnetic ordering below the Néel temperature, which, for the SmNiO_3_ samarium orthonickelate, is T_N_ ≈ 225 K, while, for the NdNiO_3_ neodymium orthonickelate, it is only T_N_ ≈ 200 K [54].

**Figure 8:**
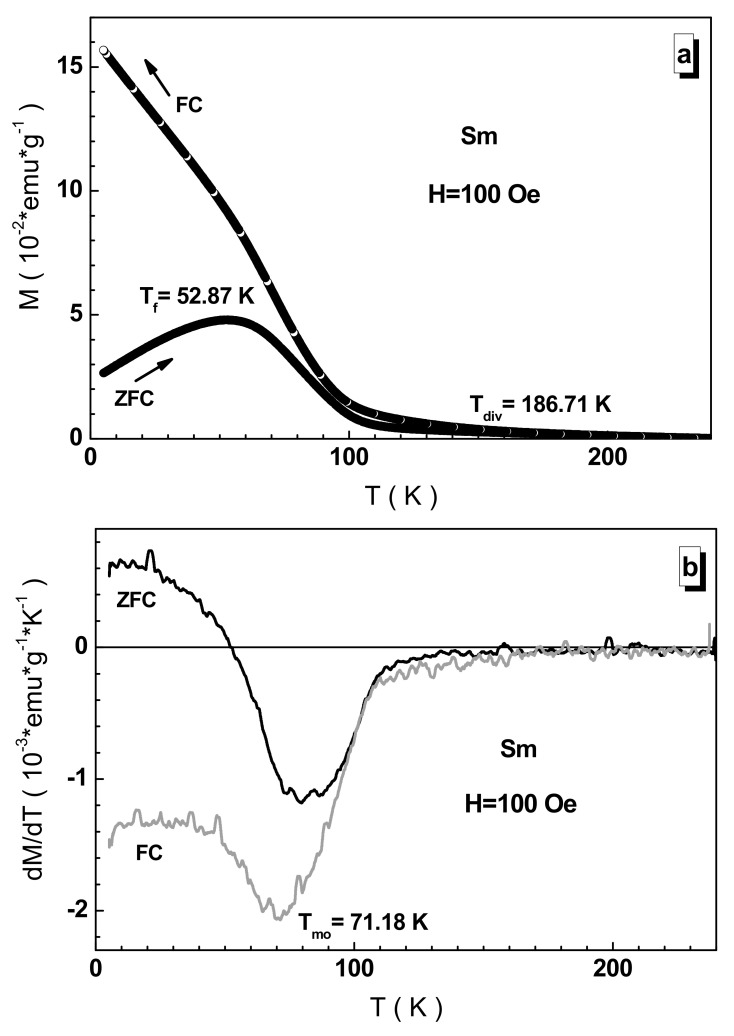
ZFC (full circle) and FC (open circle) curves of M(T) temperature magnetization (**a**) and their dM/dT(T) first magnetization derivative (**b**) for the Sm-based sample in 100 Oe. Inset demonstrates the temperature magnetization on a larger scale.

To clarify the nature of the magnetic phase state, an attempt was made to study the low-field dynamics of spins for the samples obtained. Figure 8 presents such data for the samarium sample. It is clearly seen that, depending on the magnetic prehistory, the behavior of the spins is strikingly different. The ZFC curve exhibits a maximum characteristic of the spin glass state. The T_f_ freezing temperature, which determines the average diameter of a ferromagnetically ordered inclusion in a paramagnetic or antiferromagnetic medium, is ~53 K. According to the Bean–Livingstone ratio [55], this freezing temperature corresponds to the <D> average diameter of a ferromagnetically ordered inclusion equal to ~50 nm. The T_div_ divergence temperature of the ZFC and FC curves, which determines the D_max_ maximum diameter of a ferromagnetically ordered inclusion, is ~187 K. It corresponds to the diameter of the maximum ferromagnetically ordered inclusion equal to ~180 nm. These data correlate well for the same previously obtained type of high-entropy perovskites based on such rare-earth cations as La^3+^ and Nd^3+^. It should be noted that the magnetic ordering temperature in a weak field is much lower and is only ~71 K for the samarium sample (see Figure 8b).

**Figure 9:**
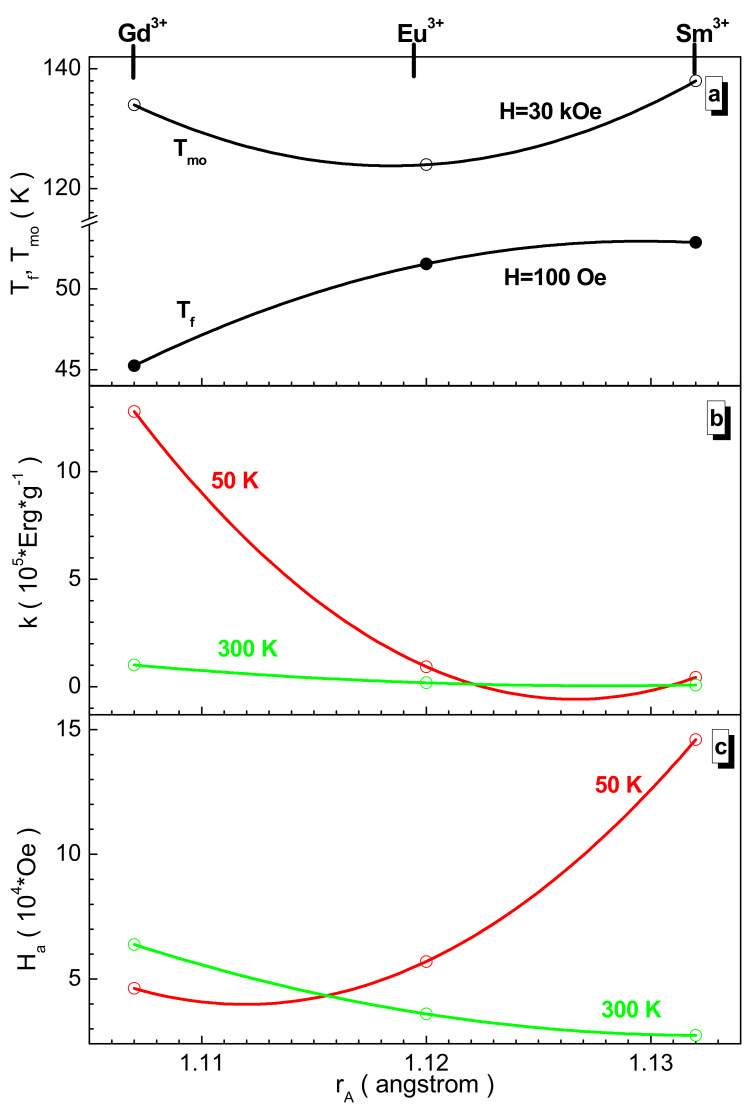
Dependence of the T_mo_ magnetic ordering (in 30 kOe) and T_f_ freezing (in 100 Oe) temperature (**a**), k magnetic crystallographic anisotropy coefficient (**b**), and H_a_ anisotropy field (**c**) vs. A-site cation radius size at 50 K (red circle and line) and 300 K (green circle and line) for the obtained samples. The solid line is the second-order polynomial interpolation and it is an eye guide.

Figure 9 presents a summary of critical temperatures such as the freezing and magnetic ordering ones and the calculated magnetic parameters such as the k magnetic crystallographic anisotropy coefficient and anisotropy field. It can be seen that the critical temperatures have an oppositely directed behavior depending on the size of the rare-earth cation. The freezing temperature decreases monotonically from ~53 K for the samarium sample and decreases by ~14% for the gadolinium sample. At the same time, the magnetic ordering temperature passes through a local minimum for the europium sample, for which it is equal to ~124 K. The relative decrease in this temperature for the samarium sample from ~138 K to the gadolinium sample down to ~134 K is ~3%. For these temperatures, the corresponding average and maximum diameter of the ferromagnetic nanoinclusions are calculated, and they are in the range of 40–50 nm and 160–180 nm, respectively. The nonmonotonic behavior of the magnetic ordering temperature for the polysubstituted perovskites looks somewhat unusual, as, for each individual 3d cation [56], an increase in the magnetic ordering temperature, as a rule, the Néel temperature, and an increase in the spontaneous magnetization are observed as the serial number of the rare-earth cation increases [57].

Very often, in order to find materials with the required microwave parameters, it is necessary to know and optimize the anisotropic characteristics [58]. The magnetic properties of the high-entropy perovskite solid solutions obtained in this work will make it possible to select the required anisotropic parameters for the optimization of microwave materials in the future. This is the task of research in the near future.

In the approximation of uniaxial anisotropy, the calculation is quite simple [59]. Such data are presented in Figure 9b,c. The k magnetic crystallographic anisotropy coefficient increases almost monotonically as the seral number of the rare-earth cation increases at both temperatures. Thus, at 50 K, it increases from ~4.37 × 10^4^ erg/g for the samarium sample by ~193% for the gadolinium sample, and at 300 K, it increases from ~8.09 × 10^3^ erg/g for the samarium sample by ~1152% for the gadolinium sample. The H_a_ anisotropy field behaves in different directions at different temperatures [60,61,62]. Thus, at 50 K, it falls from ~14.6 × 10^4^ Oe for the samarium sample by ~68% for the gadolinium sample, while, at 300 K, it increases from ~2.74 × 10^4^ Oe for the samarium sample by ~133% for the gadolinium sample. This change in the behavior of the anisotropy field is explained by a sharp decrease in the spontaneous magnetization, which is dominant [63,64,65].

From the analysis of the above experimental information and already known literature data, it can be concluded that the investigated Sm(Eu,Gd)Cr_0.2_Mn_0.2_Fe_0.2_Co_0.2_Ni_0.2_O_3_ poly-substituted high-entropy perovskites are inhomogeneous antiferromagnets characterized by a magnetic state similar to the state of a spin glass with nanosized ferromagnetically ordered inclusions in an antiferromagnetic or paramagnetic medium and weak magnetic characteristics [66]. The complex substitution of B-cations in the perovskite cell increases the phase stability of the studied solid solutions, but decreases the magnetic parameters due to magnetic dilution and weakening of the Mn^3+^(Cr^3+^, Fe^3+^, Co^3+^, Ni^2+^)-O^2^-Mn^3+^(Cr^3+^, Fe^3+^, Co^3+^, Ni^2+^) indirect superexchange interactions. Reducing the size of the rare-earth cation has a stabilizing role due to the effect of chemical compression of the unit cell.

## 4. Conclusions

Three high-entropy Sm(Eu,Gd)Cr_0.2_Mn_0.2_Fe_0.2_Co_0.2_Ni_0.2_O_3_ perovskite solid solutions were synthesized using the usual ceramic technology. The XRD investigation at room temperature established the single-phase perovskite product. The Rietveld refinement with the FullProf computer program in the frame of the orthorhombic Pnma (No 62) space group was realized. Along with a decrease in the V unit cell volume from ~224.33 Å^3^ for the Sm-based sample down to ~221.52 Å^3^ for the Gd-based sample, an opposite tendency was observed for the unit cell parameters as the ordinal number of the rare-earth cation increased. The a and c unit cell parameters decreased from ~5.352 Å (Sm) and ~5.403 Å (Gd) down to ~5.293 Å (Sm) and ~5.367 Å (Gd), respectively, while the c unit cell parameter increased from ~5.502 Å (Sm) up to ~5.530 Å (Gd). The average grain size was in the range of 5–8 μm. Field magnetization was measured up to 30 kOe at 50 K and 300 K. The law of approach to saturation was used to determine the M_s_ spontaneous magnetization, which nonlinearly increased from ~1.89 emu/g (Sm) up to ~17.49 emu/g (Gd) and from ~0.59 emu/g (Sm) up to ~3.16 emu/g (Gd) at 50 K and 300 K, respectively. The M_r_ residual magnetization and H_c_ coercive force were also determined, while the SQR loop squareness, k magnetic crystallographic anisotropy coefficient, and H_a_ anisotropy field were calculated. The M_r_ residual magnetization nonlinearly increased from ~0.19 emu/g (Sm) up to ~0.72 emu/g (Gd) at 50 K. The H_c_ coercive force nonlinearly decreased from ~2.80 Oe (Sm) down to ~ 0.93 Oe (Gd) at 50 K. Temperature magnetization was measured in field of 30 kOe. ZFC and FC magnetization curves were fixed in a field of 100 Oe. It was discovered that the T_mo_ magnetic ordering temperature downward-curve decreased from ~137.98 K (Sm) down to ~133.99 K (Gd) in 30 kOe, while the T_f_ freezing temperature, on the contrary, upward-curve decreased from ~52.87 K (Sm) down to ~45.25 K. The spin glass state with ferromagnetic nanoinclusions for all the samples was observed. The <D> average and D_max_ maximum diameter of ferromagnetic nanoinclusions were calculated, and they were in the range of 40–50 nm and 160–180 nm, respectively. The mechanism of magnetic state formation was discussed in terms of the effects of the A-site cation size and B-site poly-substitution on the Mn^3+^(Cr^3+^, Fe^3+^, Co^3+^, Ni^2+^)-O^2−^-Mn^3+^(Cr^3+^, Fe^3+^, Co^3+^, Ni^2+^) indirect superexchange interactions.

## Data Availability

Not applicable. Data could be shared upon reasonable request.
